# Supplementary material for: Ligand-Dependent Opening of the Multiple AMPA Receptor Conductance States: A Concerted Model
Source: PLoS One. 2015 Jan 28;10(1):e0116616. doi: 10.1371/journal.pone.0116616 (PMC4309570; doi:10.1371/journal.pone.0116616)
Supplement: S1 Supporting equations — (PDF) [file pone.0116616.s001.pdf]

Supplementary equations

Ranjita Dutta Roy

June 2, 2014

1 Definitions

$$B = B_0 + \frac{4B_0X}{K_B} + \frac{6B_0X^2}{K_B^2} + \frac{4B_0X^3}{K_B^3} + \frac{B_0X^4}{K_B^4} \tag{1}$$

$$S = A_0^S + \frac{4A_0^SX}{K_S} + \frac{6A_0^SX^2}{K_s^2} + \frac{4A_0^SX^3}{K_s^3} + \frac{A_0^SX^4}{K_s^4} \tag{2}$$

$$M = A_0^M + \frac{4A_0^MX}{K_M} + \frac{6A_0^MX^2}{K_M^2} + \frac{4A_0^MX^3}{K_M^3} + \frac{A_0^MX^4}{K_M^4} \tag{3}$$

$$L = A_o^L + \frac{4A_0^LX}{K_L} + \frac{6A_0^LX^2}{K_L^2} + \frac{4A_0^LX^3}{K_L^3} + \frac{A_0^LX^4}{K_L^4} \tag{4}$$

$$\frac{B}{A_0^L} = L_SL_ML_L + \frac{4L_SL_ML_LX}{K_B} + \frac{6L_SL_ML_LX^2}{K_B^2} + \frac{4L_SL_ML_LX^3}{K_B^3} + \frac{L_SL_ML_LX^4}{K_B^4} \tag{5}$$

$$\frac{S}{A_0^L} = L_ML_L + \frac{4L_ML_LX}{K_S} + \frac{6L_ML_LX^2}{K_S^2} + \frac{4L_ML_LX^3}{K_S^3} + \frac{L_ML_LX^4}{K_S^4} \tag{6}$$

$$\frac{M}{A_0^L} = L_L + \frac{4L_LX^2}{K_M} + \frac{6L_LX^2}{K_M^2} + \frac{4L_LX^3}{K_M^3} + \frac{L_LA_0^LX^4}{K_M^4} \tag{7}$$

$$\frac{L}{A_0^L} = 1 + \frac{4X}{K_L} + \frac{6X^2}{K_L^2} + \frac{4X^3}{K_L^3} + \frac{X^4}{K_L^4} \tag{8}$$

2 Derivation of the saturation function

$$Y = \frac{B_1 + 2B_2 + 3B_3 + 4B_4 + A_1^S + 2A_2^S + 3A_3^S + 4A_4^S + A_1^M + 2A_2^M + 3A_3^M + 4A_4^M + A_1^L + 2A_2^L + 3A_3^L + 4A_4^L}{4(B_0 + B_1 + B_2 + B_3 + B_4 + A_0^S + A_1^S + A_2^S + A_3^S + A_4^S + A_0^M + A_1^M + A_2^M + A_3^M + A_4^M + A_0^L + A_1^L + A_2^L + A_3^L + A_4^L)} \tag{9}$$

$$Y = \frac{B_0\frac{X}{K_B}(1+\frac{X}{K_B})^3 + A_0^S\frac{X}{K_S}(1+\frac{X}{K_S})^3 + A_0^M\frac{X}{K_M}(1+\frac{X}{K_M})^3 + A_0^L\frac{X}{K_L}(1+\frac{X}{K_L})^3}{B_0\frac{X}{K_B}(1+\frac{X}{K_B})^4 + A_0^S\frac{X}{K_S}(1+\frac{X}{K_S})^3 + A_0^M\frac{X}{K_M}(1+\frac{X}{K_M})^3 + A_0^L\frac{X}{K_L}(1+\frac{X}{K_L})^3} \tag{10}$$

$$Y = \frac{B_0 \frac{X}{K_B}(1+\frac{X}{K_B})^3 + \frac{B_0}{L_S}\frac{X}{K_S}(1+\frac{X}{K_S})^3 + \frac{B_0}{L_M}\frac{X}{K_M}(1+\frac{X}{K_M})^3 + \frac{B_0}{L_L}\frac{X}{K_L}(1+\frac{X}{K_L})^3}{B_0 \frac{X}{K_B}(1+\frac{X}{K_B})^4 + \frac{B_0}{L_S}\frac{X}{K_S}(1+\frac{X}{K_S})^4 + \frac{B_0}{L_M}\frac{X}{K_M}(1+\frac{X}{K_M})^4 + \frac{B_0}{L_L}\frac{X}{K_L}(1+\frac{X}{K_L})^4} \tag{11}$$

$$Y = \frac{B_0 \frac{X}{K_B}(1+\frac{X}{K_B})^3 + \frac{1}{L_S}\frac{X}{K_S}(1+\frac{X}{K_S})^3 + \frac{1}{L_M}\frac{X}{K_M}(1+\frac{X}{K_M})^3 + \frac{1}{L_L}\frac{X}{K_L}(1+\frac{X}{K_L})^3}{\frac{X}{K_B}(1+\frac{X}{K_B})^4 + \frac{1}{L_S}\frac{X}{K_S}(1+\frac{X}{K_S})^4 + \frac{1}{L_M}\frac{X}{K_M}(1+\frac{X}{K_M})^4 + \frac{1}{L_L}\frac{X}{K_L}(1+\frac{X}{K_L})^4} \tag{12}$$

3 Derivation of a state function, example of the large conductance

$$A_L = \frac{A_o^L + A_1^L + A_2^L + A_3^L + A_4^L}{B_0 + B_1 + B_2 + B_3 + B_4 + A_o^s + A_1^L + A_2^s + A_3^s + A_4^s + A_o^M + A_1^M + A_2^M + A_3^M + A_4^M + A_o^L + A_1^L + A_2^L + A_3^L + A_4^L} \tag{13}$$

$$A_L = \frac{A_o^L + \frac{4A_0^L X}{K_L} + \frac{6A_0^L X^2}{K_L^2} + \frac{4A_0^L X^3}{K_L^3} + \frac{A_0^L X^4}{K_L^4}}{B + S + M + L} \tag{14}$$

$$A_L = \frac{1 + \frac{4X}{K_L} + \frac{6X^2}{K_L^2} + \frac{4X^3}{K_L^3} + \frac{X^4}{K_L^4}}{(B + S + M + L)/A_0^L} \tag{15}$$

$$A_L = \frac{(1+\frac{X}{K_L})^4}{L_S L_M L_L (1+\frac{X}{K_B})^4 + L_M L_L (1+\frac{X}{K_S})^4 + L_L (1+\frac{X}{K_M})^4 + (1+\frac{X}{K_L})^4} \tag{16}$$

4 Kinetic model

$$\frac{d[B_0]}{dt} = -4^B k_{on}[B_0][G] +^B k_{off}[B_1] -^{BS} k_0[B_0] +^{SB} k_0[A_0^S] \tag{17}$$

$$\frac{d[B_1]}{dt} = -3^B k_{on}[B_1][G] + 2^B k_{off}[B_2] -^B k_{off}[B_1] + 4^B k_{on}[B_0][G] -^{BS} k_1[B_1] +^{SB} k_1[A_1^S] \tag{18}$$

$$\frac{d[B_2]}{dt} = -2^B k_{on}[B_2][G] + 3^B k_{off}[B_3] - 2^B k_{off}[B_2] + 3^B k_{on}[B_1][G] -^{BS} k_2[B_2] +^{SB} k_2[A_2^S] \tag{19}$$

$$\frac{d[B_3]}{dt} = -^B k_{on}[B_3][G] + 4^B k_{off}[B_4] - 3^B k_{off}[B_3] + 2^B k_{on}[B_2][G] -^{BS} k_3[B_3] +^{SB} k_3[A_3^S] \tag{20}$$

$$\frac{d[B_4]}{dt} = -4^B k_{off}[B_4] +^B k_{on}[B_3][G] -^{BS} k_4[B_4] +^{SB} k_3[A_4^S] \tag{21}$$

$$\frac{d[A_0^S]}{dt} = -4^S k_{on}[A_0^S][G] +^S k_{off}[A_1^S] -^{SM} k_0[A_0^S] +^{MS} k_0[A_0^M] -^{SB} k_0[A_0^S] +^{BS} k_0[B_0] \tag{22}$$

$$\frac{d[A_1^S]}{dt} = -3^S k_{on}[A_1^S][G] + 2^S k_{off}[A_2^S] -^S k_{off}[A_1^S] + 4^S k_{on}[A_0^S][G] -^{SM} k_1[A_1^S] +^{MS} k_1[A_1^M] -^{SB} k_1[A_1^M] +^{BS} k_1[B_1] \tag{23}$$

$$\frac{d[A_2^S]}{dt} = -2^S k_{on}[A_2^S][G] + 3^S k_{off}[A_3^S] - 2^S k_{off}[A_2^S] + 3^S k_{on}[A_1^S][G] -^{SM} k_2[A_2^S] +^{MS} k_2[A_2^M] -^{SB} k_2[A_2^M] +^{BS} k_2[B_2] \tag{24}$$

$$\frac{d[A_3^S]}{dt} = -^S k_{on}[A_3^S][G] + 4^S k_{off}[A_4^S] - 3^S k_{off}[A_3^S] + 2^S k_{on}[A_2^S][G] -^{SM} k_3[A_3^S] +^{MS} k_3[A_3^M] -^{SB} k_3[A_3^M] +^{BS} k_3[B_3] \tag{25}$$

$$\frac{d[A_4^S]}{dt} = -4^S k_{off}[A_4^S] + ^S k_{on}[A_3^S][G] - ^{SM} k_4[A_4^S] + ^{MS} k_4[A_4^M] - ^{SB} k_4[A_4^M] + ^{BS} k_4[B_4] \tag{26}$$

$$\frac{d[A_0^M]}{dt} = -4^M k_{on}[A_0^M][G] + ^M k_{off}[A_1^M] - ^{ML} k_0[A_0^M] + ^{LM} k_0[A_0^L] - ^{MS} k_0[A_0^M] + ^{SM} k_0[A_0^S] \tag{27}$$

$$\frac{d[A_1^M]}{dt} = -3^M k_{on}[A_1^M][G] + 2^M k_{off}[A_2^M] - ^M k_{off}[A_1^M] + 4^M k_{on}[A_0^M][G] - ^{ML} k_1[A_1^M] + ^{LM} k_1[A_1^L] - ^{MS} k_1[A_1^M] + ^{SM} k_1[A_1^S] \tag{28}$$

$$\frac{d[A_2^M]}{dt} = -2^M k_{on}[A_2^M][G] + 3^M k_{off}[A_3^M] - 2^M k_{off}[A_2^M] + 3^M k_{on}[A_1^M][G] - ^{ML} k_2[A_2^M] + ^{LM} k_2[A_2^L] - ^{MS} k_2[A_2^M] + ^{SM} k_2[A_2^S] \tag{29}$$

$$\frac{d[A_3^M]}{dt} = -^M k_{on}[A_3^M][G] + 4^M k_{off}[A_4^M] - 3^M k_{off}[A_3^M] + 2^M k_{on}[A_2^M][G] - ^{ML} k_3[A_3^M] + ^{LM} k_3[A_3^L] - ^{MS} k_3[A_3^M] + ^{SM} k_3[A_3^S] \tag{30}$$

$$\frac{d[A_4^M]}{dt} = -4^M k_{off}[A_4^M] + ^M k_{on}[A_3^M][G] - ^{ML} k_4[A_4^M] + ^{LM} k_4[A_4^L] - ^{MS} k_4[A_4^M] + ^{SM} k_4[A_4^S] \tag{31}$$

$$\frac{d[A_0^L]}{dt} = -4^L k_{on}[A_0^L][G] + ^L k_{off}[A_1^L] - ^{LM} k_0[A_0^L] + ^{ML} k_0[A_0^M] \tag{32}$$

$$\frac{d[A_1^L]}{dt} = -3^L k_{on}[A_1^L][G] + 2^L k_{off}[A_2^L] - ^L k_{off}[A_1^L] + 4^L k_{on}[A_0^L][G] - ^{LM} k_1[A_1^L] + ^{ML} k_1[A_1^M] \tag{33}$$

$$\frac{d[A_2^L]}{dt} = -2^L k_{on}[A_2^L][G] + 3^L k_{off}[A_3^L] - 2^L k_{off}[A_2^L] + 3^L k_{on}[A_1^L][G] - ^{LM} k_2[A_2^L] + ^{ML} k_2[A_2^M] \tag{34}$$

$$\frac{d[A_3^L]}{dt} = -^L k_{on}[A_3^L][G] + 4^L k_{off}[A_4^L] - 3^L k_{off}[A_3^L] + 2^L k_{on}[A_2^L][G] - ^{LM} k_3[A_3^L] + ^{ML} k_3[A_3^M] \tag{35}$$

$$\frac{d[A_4^L]}{dt} = -4^L k_{off}[A_4^L] + ^L k_{on}[A_3^L][G] - ^{LM} k_4[A_4^L] + ^{ML} k_4[A_4^M] \tag{36}$$
